# Emotion Regulation Training May Improve Stress, Depression, Anxiety, and Physical Activity

**DOI:** 10.1093/geroni/igaa057.1967

**Published:** 2020-12-16

**Authors:** Kelly Wierenga, David Fresco, Megan Alder, Shirley Moore

**Affiliations:** 1 Indiana University, Indianapolis, Indiana, United States; 2 University of Michigan, Ann Arbor, Michigan, United States; 3 Case Western Reserve University, Cleveland, Ohio, United States; 4 Case Western Reserve University, Shaker Heights, Ohio, United States

## Abstract

The purpose of this two-arm randomized controlled pilot study was to assess initial efficacy of the theoretically-based RENEwS intervention, designed to improve emotion regulation and thereby decrease depression and anxiety and increase moderate to vigorous physical activity (MVPA) following a cardiac event. Participants (n=30, 83% men) recruited from cardiac rehabilitation were randomized to five weekly 1-hour sessions of RENEwS intervention or active control. Although this trial was not powered for confirmatory efficacy (p’s > .02, but many greater than .05), RENEwS participants evidenced an advantage over Control participants in terms of reductions in stress (Cohen’s f = .47), depression symptoms (Cohen’s f = .34), anxiety symptoms (Cohen’s f = .40) but only modest improvements in MVPA from baseline to 5 months (Cohen’s f = .08). Findings support potential efficacy and testing RENEwS in a larger sample.

